# Activation of cAMP Signaling in Response to α-Phellandrene Promotes Vascular Endothelial Growth Factor Levels and Proliferation in Human Dermal Papilla Cells

**DOI:** 10.3390/ijms23168959

**Published:** 2022-08-11

**Authors:** Wesuk Kang, Soyoon Park, Dabin Choi, Bomin Son, Taesun Park

**Affiliations:** Department of Food and Nutrition, BK21 FOUR, Yonsei University, 50 Yonsei-ro, Seodaemun-gu, Seoul 120-749, Korea

**Keywords:** cAMP, α-phellandrene, vascular endothelial growth factor, cell proliferation, dermal papilla cells

## Abstract

Dermal papilla cells (DPCs) are growth factor reservoirs that are specialized for hair morphogenesis and regeneration. Due to their essential role in hair growth, DPCs are commonly used as an in vitro model to investigate the effects of hair growth-regulating compounds and their molecular mechanisms of action. Cyclic adenosine monophosphate (cAMP), an intracellular second messenger, is currently employed as a growth-promoting target molecule. In a pilot test, we found that α-phellandrene, a naturally occurring phytochemical, increased cAMP levels in DPCs. Therefore, we sought to determine whether α-phellandrene increases growth factors and proliferation in human DPCs and to identify the underlying mechanisms. We demonstrated that α-phellandrene promotes cell proliferation concentration-dependently. In addition, it increases the cAMP downstream effectors, such as protein kinase A catalytic subunit (PKA Cα) and phosphorylated cAMP-responsive element-binding protein (CREB). Also, among the CREB-dependent growth factor candidates, we identified that α-phellandrene selectively upregulated vascular endothelial growth factor (VEGF) mRNA expression in DPCs. Notably, the beneficial effects of α-phellandrene were nullified by a cAMP inhibitor. This study demonstrated the cAMP-mediated growth effects in DPCs and the therapeutic potential of α-phellandrene for preventing hair loss.

## 1. Introduction

Hair loss is a result of hair thinning. The dermal papilla (DP), which is located at the base of the hair follicle, plays a crucial role in regulating hair shaft thickness [1,2,3]. The roles of growth factors in this process have received considerable attention recently. Growth factors can stimulate the cells surrounding the hair follicle, such as the endothelial and epithelial cells, to vigorously proliferate and differentiate, which could contribute to thickening hair shafts and promoting hair growth [4,5,6]. Although hair follicles are composed of more than 20 types of cells, DP cells (DPCs) are generally regarded as a key player in growth factor production and supply to the hair follicle [1,7,8,9]. These growth factors also stimulate the producer cell itself to proliferate rapidly, which could ultimately result in growth factor regeneration. Therefore, increasing growth factor secretion in DPCs has been considered a promising strategy to prevent and treat hair loss [10,11,12,13,14].

Therapeutic targets for boosting growth factors have been studied continuously for several decades. G-protein-coupled receptors (GPCRs), which are located on the cell surface, have gained pharmacological interest as an attractive molecular target due to their ubiquity and easy accessibility to extracellular molecules. Indeed, the vast majority of the US Food and Drug Administration (FDA)-approved drugs (~40%) exert their effects by activating the GPCR-mediated cyclic adenosine monophosphate (cAMP) pathway [15,16]. For example, adrenergic receptor agonist-induced cAMP accumulation enhances vascular endothelial growth factor (VEGF) gene expression in various cells, including brown adipocytes, cardiac myocytes, and macrophages [17,18,19]. We have previously reported that elevated intracellular cAMP levels by several phytochemicals (e.g., cedrene and nonanal) upregulate several growth factors, including insulin-like growth factor-1 (IGF-1) in myoblasts and DPCs, which is significantly ameliorated by cAMP inhibitors [20,21]. Although it has not been clearly established, several recent studies have shown that cAMP-dependent growth factor gene expression regulation is potentially associated with protein kinase A (PKA) and its downstream effectors, cAMP-responsive element-binding protein (CREB) [20,22,23].

Currently, two synthetic chemicals, minoxidil and finasteride, are exclusively approved by the FDA and considered the main options for treating hair loss. However, due to their side effects, including eczema, ejaculatory dysfunction, and cognitive symptoms, there has been steady progress in the development of novel hair growth promoters from natural compounds [24,25,26]. We attempted to find a potential biocontrol agent that has been empirically applied in several fields, such as cosmetics and food additives. α-Phellandrene, found in essential oils such as eucalyptus, lavender, and geranium, is approved as a safe flavoring agent by credible organizations, including the FDA, the Joint Food and Agriculture Organization of United Nations (FAO)/World Health Organization (WHO) Expert Committee on Food Additives (JECFA), and Flavor Extract Manufacturers Association (FEMA). It has been widely used in commercial applications, such as cosmetics, detergents, and perfumes because of its characteristic aroma. It is also regarded as a bioactive molecule receiving considerable scientific attention due to its broad-spectrum physiological functions, such as anti-nociceptive, anti-inflammatory, and anti-antimicrobial effects in vitro and in vivo [27,28,29,30]. This study aimed to determine whether α-phellandrene activates the cAMP signaling pathway and whether this compound influences proliferation and growth factors via the cAMP pathway in DPCs.

## 2. Results

### 2.1. α-Phellandrene Increases cAMP Levels in DPCs

**Figure 1:**
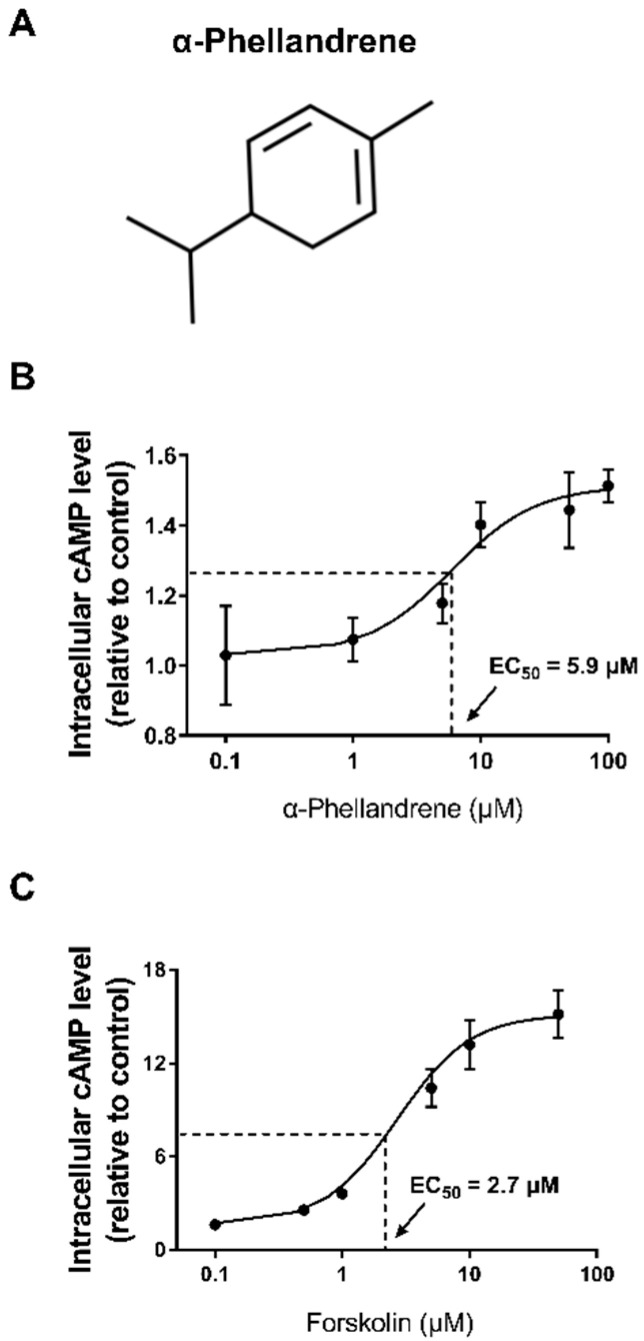
α-phellandrene increases cyclic adenosine monophosphate (cAMP) levels in dermal papilla cells (DPCs). (**A**) The structure of α-phellandrene. (**B**,**C**) Total cAMP content was measured in DPCs after 30 min stimulation with vehicle (dimethyl sulfoxide; DMSO) or various concentrations of α-phellandrene or forskolin. Results are expressed as mean ± standard error of the mean (SEM) of three independent experiments.

cAMP signaling can promote cell proliferation; thus, we attempted to identify the molecules that increase cAMP levels in DPCs. We found that α-phellandrene significantly increased cAMP levels in a concentration-dependent manner, with the half-maximal effective concentration (EC_50_) being 5.9 μM (Figure 1A,B). Additionally, we confirmed that forskolin, a well-known cAMP stimulator, also induced cAMP accumulation in a dose-dependent manner (Figure 1C).

### 2.2. α-Phellandrene Promotes DPC Proliferation

**Figure 2:**
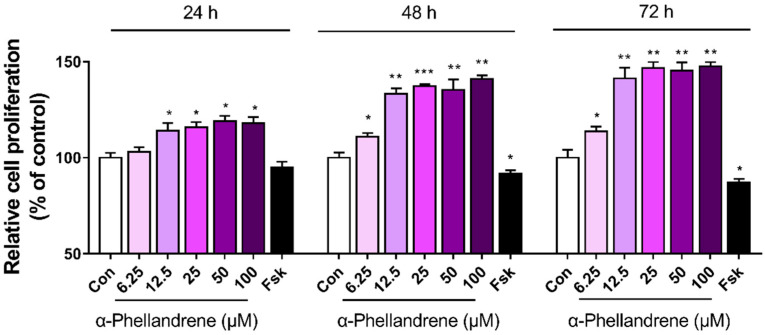
α-phellandrene promotes cell proliferation in dermal papilla cells (DPCs). DPCs were cultured for 24, 48, and 72 h with vehicle (dimethyl sulfoxide; DMSO) as a control (Con), five different α-phellandrene concentrations (6.25, 12.5, 25, 50, and 100 µM), or 10 µM forskolin (Fsk). Cell proliferation was measured using a water-soluble tetrazolium salt (WST-1). Results are shown as mean ± standard error of the mean (SEM) of three independent experiments. Statistically significant differences are marked as * *p* < 0.05 and ** *p* < 0.01, *** *p* < 0.001 vs. control (Con).

We further determined whether α-phellandrene stimulates DPC proliferation at 24, 48, and 72 h after treatment using a Water-Soluble Tetrazolium Salt (WST-1) assay. Considering that α-phellandrene exhibited potent cAMP-elevating activity with EC_50_ of 5.9, and that the EC_50_ value is often used as a reference for the critical concentration of chemical compounds required for their significant action, we hypothesized that its physiological effects may begin to manifest at least over 5.9 μM. Thus, DPCs were cultured in the presence of vehicle control (dimethyl sulfoxide; DMSO) or five different concentrations of α-phellandrene (6.25, 12.5, 25, 50, and 100 µM). α-phellandrene significantly increased DPC proliferation in a concentration-dependent manner, and this effect appeared to be saturated at 12.5 µM. On the other hand, forskolin treatment (10 µM) significantly decreased DPC proliferation (Figure 2). To investigate the effect of α-phellandrene on cell death, the DPCs were treated with either vehicle control (DMSO) or various concentrations of α-phellandrene (12.5–1200 μM). Treatment of α-phellandrene at concentrations as high as 600 μM did not cause cell death, and cytotoxicity was observed to occur after treatment with 800 μM or more of α-phellandrene (Figure S1).

### 2.3. cAMP Accumulation by α-Phellandrene Leads to the Stimulation of Downstream PKA/CREB Signaling in DPCs

**Figure 3:**
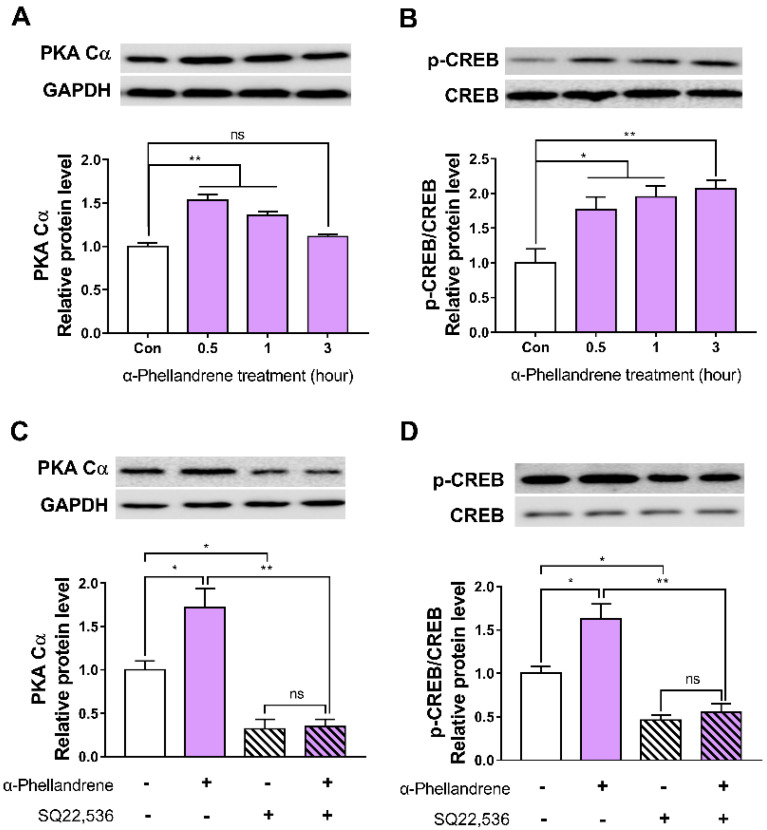
α-phellandrene-mediated cyclic adenosine monophosphate (cAMP) accumulation stimulates the downstream protein kinase (PKA)/cAMP-responsive element-binding protein (CREB) signaling in dermal papilla cells (DPCs). DPCs were treated with vehicle (dimethyl sulfoxide; DMSO) or 12.5 μM α-phellandrene for 0.5, 1, and 3 h, following which (**A**) protein kinase A catalytic subunit (PKA Cα) expression and (**B**) CREB phosphorylation were quantified by western blotting. Cells were incubated with either vehicle or 12.5 μM of α-phellandrene for 0.5 h. The cells were preincubated with the vehicle or 50 μM of SQ22,536 (cAMP inhibitor) for 1 h before α-phellandrene treatment. (**C**) PKA Cα expression and (**D**) CREB phosphorylation were quantified by western blotting. Relative PKA Cα protein expression was normalized to glyceraldehyde 3-phosphate dehydrogenase (GAPDH) level and the ratio of CREB phosphorylation normalized to total CREB is shown (*p*-CREB/CREB). Results are expressed as mean ± standard error of the mean (SEM) of three independent experiments. Statistically significant differences are marked as ns, not significant (*p* > 0.05); * *p* < 0.05; ** *p* < 0.01.

We also examined whether α-phellandrene regulates the cAMP downstream effectors, such as PKA catalytic subunit (PKA Cα) and phosphorylated CREB in DPCs. α-phellandrene upregulated both PKA Cα expression and CREB phosphorylation level in a time-dependent manner, and significant increases were observed after 0.5 h (Figure 3A,B).

To further investigate whether the changes in PKA/CREB signaling were involved in the α-phellandrene-induced increase in cAMP levels, we measured PKA Cα expression and CREB phosphorylation level in the presence of 50 µM of SQ22,536. Exposure to SQ22,536 significantly decreased PKA Cα expression and CREB phosphorylation level in α-phellandrene-treated DPCs (Figure 3C,D).

### 2.4. α-Phellandrene Promotes Cell Proliferation via the cAMP Signaling Pathway

**Figure 4:**
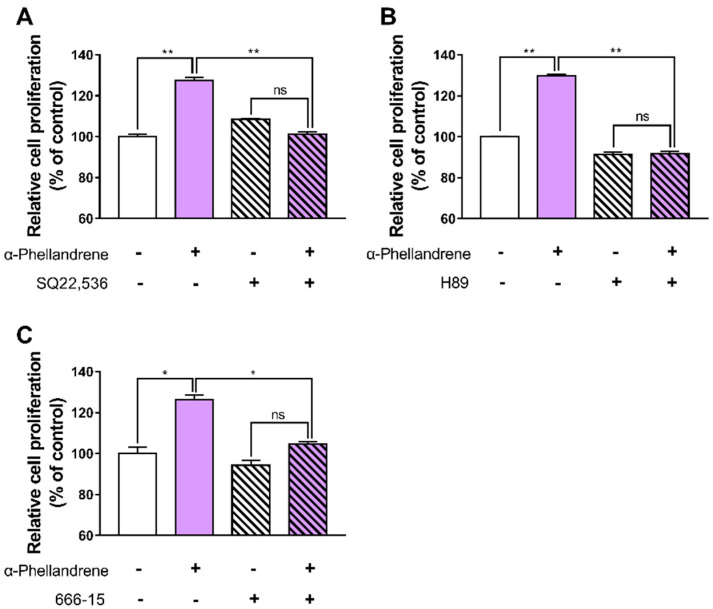
α-phellandrene promotes cell proliferation via the cyclic adenosine monophosphate (cAMP) signaling pathway. Cells were incubated with either vehicle (dimethyl sulfoxide; DMSO) or 12.5 μM of α-phellandrene for 48 h. The cells were preincubated with the vehicle, 50 μM of SQ22,536, 10 μM of H89 (protein kinase A inhibitor), or 100 nM of 666-15 (cAMP-responsive element-binding protein inhibitor) for 1 h before α-phellandrene treatment. (**A**–**C**) Cell proliferation was measured using water-soluble tetrazolium salt (WST-1) analysis. Results are expressed as mean ± standard error of the mean (SEM) of three independent experiments. Statistically significant differences are marked as ns, not significant (*p* > 0.05); * *p* < 0.05; ** *p* < 0.01.

To exclude the possibility that α-phellandrene might promote cell proliferation through an alternative pathway, we evaluated the proliferative effect of α-phellandrene in the presence of 50 µM of SQ22,536, 10 µM of H89, or 100 nM of 666-15. All these inhibitors completely blocked α-phellandrene-induced DPC proliferation (Figure 4A–C), suggesting that α-phellandrene promotes cell growth through cAMP signaling in DPCs.

### 2.5. α-Phellandrene Increases VEGF Gene Expression in DPCs

**Figure 5:**
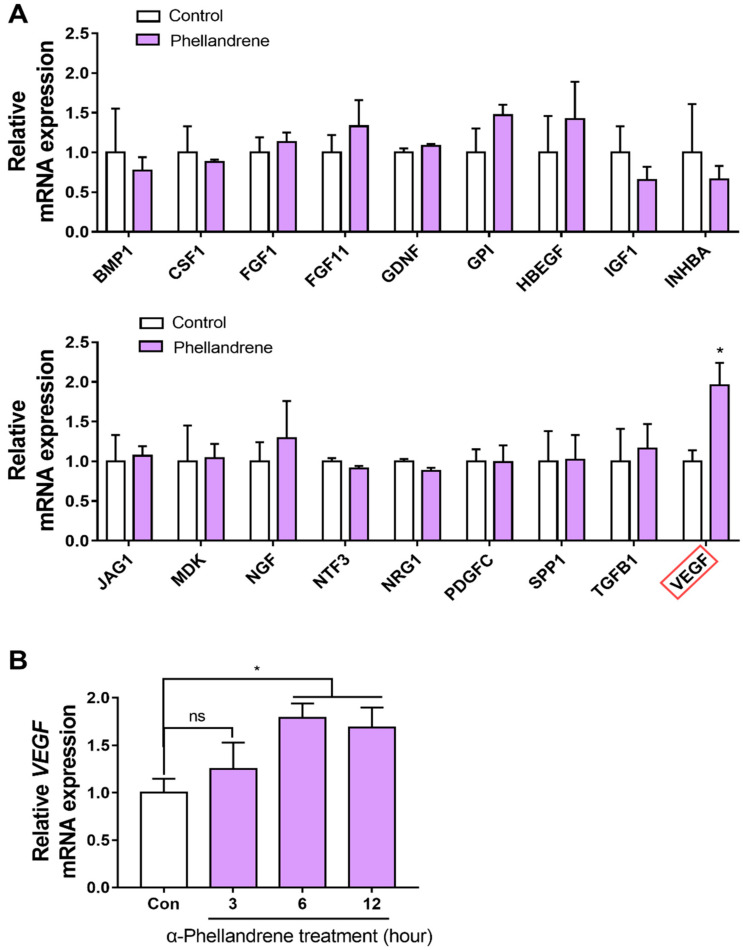
α-phellandrene increases vascular endothelial growth factor (VEGF) gene expression in dermal papilla cells (DPCs). (**A**) DPCs were either treated with vehicle (dimethyl sulfoxide; DMSO) or 12.5 μM of α-phellandrene for 6 h. Then, mRNA was extracted and quantitative polymerase chain reaction (qPCR) analyses were performed for detecting the altered expression of eighteen growth factor genes as potential target genes of cAMP-responsive element-binding (CREB) protein. Data were normalized relative to glyceraldehyde 3-phosphate dehydrogenase (GAPDH) expression in each sample and expressed relative to the control value (vehicle). Gene abbreviations are as follows: bone morphogenetic protein 1 (*BMP1*), colony stimulating factor 1(CSF1), fibroblast growth factor 1 (*FGF1*), fibroblast growth factor 11 (*FGF11*), glial cell derived neurotrophic factor (*GDNF*), glucose-6-phosphate isomerase (*GPI*), heparin binding epidermal growth factor- like growth factor (*HBEGF*), insulin-like growth factor 1 (*IGF1*), inhibin subunit beta A (*INHBA*), notch ligand jagged-1 (*JAG1*), midkine (*MDK*), nerve growth factor (*NGF*), neurotrophin 3 (*NTF3*), neuregulin 1 (*NRG1*), platelet derived growth factor C (*PDGFC*), secreted phosphoprotein 1 (*SPP1*), transforming growth factor beta 1 (*TGFB1*), and *VEGF*. The red box indicates the significantly upregulated gene in response to α-phellandrene treatment. (**B**) DPCs were either treated with vehicle or 12.5 μM of α-phellandrene and incubated for a variety of times (3, 6, and 12 h). Then VEGF mRNA expression was analyzed. Results are shown as mean ± standard error of the mean (SEM) of three independent experiments. Statistically significant differences are marked as * *p* < 0.05.

Since it is well known that the proliferation capacity of DPC is actively modulated by various growth factors, we hypothesized that α-phellandrene-induced cell proliferation is associated with the increased expression of certain growth factors. Accordingly, we listed the growth factor or growth-related genes expressed in DPCs, and then, considering that the effect of α-phellandrene was mediated through the cAMP/PKA/CREB pathway, we limited the potential genes responsible for α-phellandrene-induced cell proliferation to CREB target genes using hTFtarget (a comprehensive database for the human transcription factors and their targets). Finally, eighteen candidate genes were derived, and their mRNA expression in response to α-phellandrene treatment was analyzed [31,32,33,34,35]. These growth-related genes are as follows: bone morphogenetic protein 1 (*BMP1*), colony stimulating factor 1 (CSF1), fibroblast growth factor 1 (*FGF1*), fibroblast growth factor 11 (*FGF11*), glial cell derived neurotrophic factor (*GDNF*), glucose-6-phosphate isomerase (*GPI*), heparin binding epidermal growth factor- like growth factor (*HBEGF*), insulin-like growth factor 1 (*IGF1*), inhibin subunit beta A (*INHBA*), notch ligand jagged-1 (*JAG1*), midkine (*MDK*), nerve growth factor (*NGF*), neurotrophin 3 (*NTF3*), neuregulin 1 (*NRG1*), platelet-derived growth factor C (*PDGFC*), secreted phosphoprotein 1 (*SPP1*), transforming growth factor beta 1 (*TGFB1*), and *VEGF*. Among these, only VEGF expression significantly increased by α-phellandrene treatment (Figure 5A). We also verified that the stimulatory effect of phellandrene on VEGF mRNA expression did not show after 3 h of treatment, but became evident after 6 h of treatment and persisted after 12 h of treatment (Figure 5B).

### 2.6. The Inhibition of the cAMP Pathway Blocks the Effect of α-Phellandrene on VEGF Production

**Figure 6:**
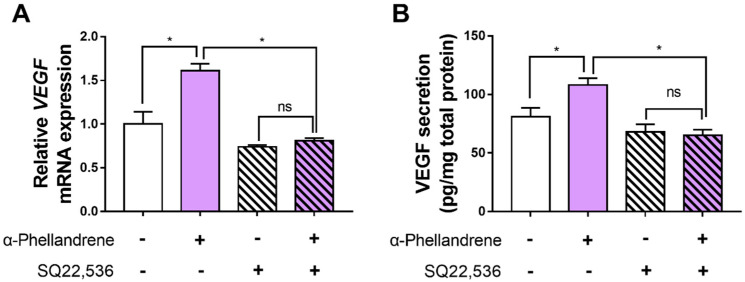
The inhibition of the cyclic adenosine monophosphate (cAMP) pathway blocks the effect of α-phellandrene on vascular endothelial growth factor (VEGF) production. (**A**,**B**) Dermal papilla cells (DPCs) were either treated with vehicle (dimethyl sulfoxide; DMSO) or 12.5 μM of α-phellandrene for 6 h (for mRNA analysis) or 24 h (for protein analysis) and pretreated with vehicle or 50 μM of SQ22,536 (cAMP inhibitor) for 1 h before α-phellandrene treatment. Then the mRNA was extracted and qPCR analyses were performed for VEGF expression changes. Each sample’s mRNA level was normalized to glyceraldehyde 3-phosphate dehydrogenase (GAPDH) and expressed relative to the value of the control (vehicle). The VEGF protein levels were normalized to the total amount of cellular protein. Results are shown as mean ± standard error of the mean (SEM) of three independent experiments. Statistically significant differences are marked as ns, not significant (*p* > 0.05); * *p* < 0.05.

**Figure 7:**
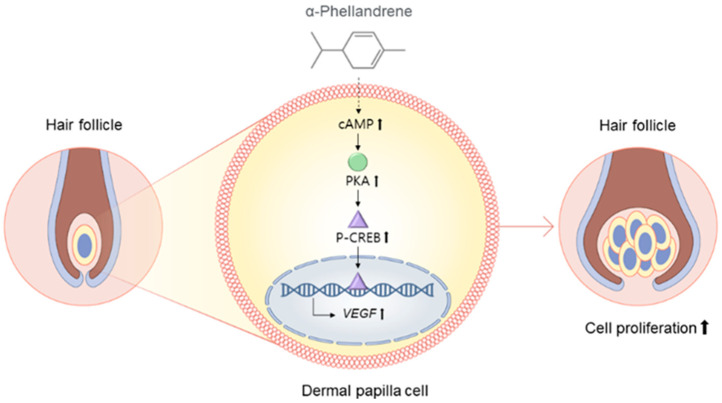
Schematic illustration of the molecular mechanism of α-phellandrene-mediated dermal papilla cell (DPC) proliferation. α-phellandrene activated the cyclic adenosine monophosphate (cAMP) signaling and increased vascular endothelial growth factor (VEGF) expression, thereby promoting DPC proliferation.

To determine whether α-phellandrene-induced VEGF production was mediated via the cAMP pathway, DPCs were treated with α-phellandrene with or without 50 µM of SQ22,536 (cAMP inhibitor). VEGF mRNA in cells was assessed and VEGF protein in media was also determined. SQ 22536 treatment completely inhibited VEGF mRNA expression in α-phellandrene-treated DPCs (Figure 6A). In line with mRNA expression, α-phellandrene significantly increased VEGF secretion from DPCs, which was blocked by SQ 22536 treatment (Figure 6B). Taken together, cAMP pathway stimulation by α-phellandrene is a possible mechanism for promoting DPC proliferation and VEGF expression (Figure 7).

## 3. Discussion

Topical delivery of agent is an efficient alternative to traditional methods, including parenteral and oral deliveries: Benefits related to the topical route include first-pass metabolism bypass, reduced systemic toxicity/adverse events, and increased bioaccessibility and bioavailability due to the proximity to the target tissue [36,37,38]. Nevertheless, the outer skin serves as a barrier for large molecules due to the presence of stratum corneum (keratin-rich corneocytes embedded in lipid bilayers); therefore, only small (typically < 500 Dalton) and lipophilic molecules penetrate the inner dermis [39]. α-phellandrene is readily absorbed and reaches the DP layer by topical route due to its low molecular size (136.2 Dalton) and lipophilicity. We confirmed that α-phellandrene is more likely to be efficiently absorbed (permeability coefficient value (log Kp): −4.85 cm/s; increased log Kp allows molecule permeation) than other well-known topical agents for alopecia (minoxidil: −7.97 cm/s; caffeine: −7.53 cm/s) and other cutaneous diseases, such as atopic dermatitis (dexamethasone: −7.32 cm/s; tacrolimus: −7.88 cm/s) using an in silico tool for predicting skin absorption [40].

In this study, we experimentally found using a specific cAMP inhibitor that α-phellandrene increased VEGF levels via the cAMP signaling pathway in DPCs. cAMP signaling is usually followed by GPCR stimulation and further adenylyl cyclase (Adcy) stimulation, which converts ATP to cAMP [41,42,43]. Although α-phellandrene has been reported to possibly bind to two GPCRs (opioid receptor and muscarinic cholinergic receptor) [27], we confirmed that these receptors are poorly expressed in basal and α-phellandrene-treated DPCs through next-generation sequencing (fragments per kilobase of transcript per million mapped reads (FPKM) = 0, unpublished data). To explore the novel upstream GPCR as a molecular target of α-phellandrene in DPCs, we employed in silico target prediction tools for predicting the possible target candidates of this specific agent for further experimental confirmation [44,45,46]. These ligand-based target identification tools have recognized several GPCRs, including adenosine receptors, dopamine receptors, and adrenergic receptors as promising candidates, which are all expressed in DPCs and considered attractive targets for the positive regulation of growth factors in mammalian cells [47,48,49]. Characterizing the specific upstream GPCR responsible for the beneficial effects of α-phellandrene in DPCs would be a promising and challenging avenue for future studies.

In the present study, two agents (forskolin and α-phellandrene) were used to compare the impact of cAMP on cell proliferation. Notably, the EC_50_ value of the α-phellandrene (5.9 μM) is as low as that of the forskolin of (2.7 μM). A low EC_50_ value indicates that the test material has a 50% effect of the maximum effects although the concentration of the test material is low. Considering that forskolin is currently accepted as the most effective cAMP activator, it would be speculated that significant effects of α-phellandrene through cAMP synthesis could be accomplished at a relatively low concentration compared to the other compounds. Also, cAMP systems act in a concentration-dependent manner, particularly in fibroblasts/fibroblast-like cells. For example, a lower cAMP level (around 150% of that in the control) in cardiac fibroblasts promotes cell migration, whereas higher cAMP levels (over 200% of that in the control) inhibit cell migration [50]. Furthermore, physiologically increased cAMP levels (150% of that in the control) activate collagen production in dermal fibroblasts, whereas extremely high cAMP levels (15,000% of that in the control), over the physiological range, inhibit collagen production [51]. In line with previous studies, a modest 12.5 µM α-phellandrene-induced cAMP increase (140% of control) promoted DPC growth, whereas a 10 µM forskolin (direct and irreversible Adcy activator)-mediated excess cAMP increase (1320% of that in the control), significantly decreased cell growth.

CREB is a well-characterized transcription factor, whose activity is regulated in various cell types by Ser133 phosphorylation. Genome-wide studies indicate that CREB binds to a target sequence named the cAMP response element (CRE) located in many cellular gene promoters and regulates up to 5000 putative target genes [52,53,54]. It is noteworthy that the capacity of CREB to bind to a specific CRE is different among the cell types; thus, the CREB target gene family differs from one cell type to another [55,56]. For example, gene profiling assays showed that exposure to forskolin, a cAMP activator, reliably induced hundreds of target genes in embryonic kidney cells as well as primary hepatocyte or pancreatic cell cultures, but the sets of cAMP-responsive genes in each case were almost completely different [57]. There is no consensus on the exact mechanism for such a cell-specific target gene activation profile, but it has been suggested that CREB binding to its target CRE is affected by the cell-specific histone methylation status at gene promoters, based on the significant correlation between specific target gene expression and histone H3 Lys-4 methylation [55]. In our study using DPCs, among the putative eighteen CREB target growth factor genes, we found that only VEGF mRNA expression was selectively upregulated by the α-phellandrene-mediated modest cAMP increase.

VEGF is an important angiogenic factor that activates hair follicle growth, which disappears during the telogen phase [58]. The hair follicles of VEGF-overexpressing mice are bigger and more numerous than those of wild mice [59]. In addition, it has been established experimentally that VEGF acts as an autocrine growth factor on human DPCs through the VEGF receptor-2 (VEGFR2) [58,60]. In line with this investigation, various studies using DPCs have shown that minoxidil and other natural agents, such as *Asiasari radix* extract, *Carthamus tinctorius* extract, and *Malva verticillata* extract, boost VEGF levels in DPCs and subsequently promote cell proliferation [61,62,63,64]. Unfortunately, in an in vitro system with one cell type, the contribution of growth factors to hair growth could not be fully represented and assessed, with cellular proliferation being the only robust phenotypic marker for assessing the effects of growth factors. Although we confirmed that α-phellandrene-mediated cAMP signaling increases VEGF levels and cell proliferation, it is well known that cAMP also promotes proliferation via multiple other mechanisms [65,66]. Further investigation is required to examine whether the enhanced VEGF levels in DPCs are solely responsible for upregulating the proliferation in response to α-phellandrene.

In contrast, in an in vivo system, growth factors can play additional roles in preventing and treating hair loss in peripheral target tissues. Notably, VEGF can function in the epidermis, where it promotes keratin generation in hair via the enhancement of keratinocyte proliferation, differentiation, and migration [67]. In addition, VEGF not only acts on the keratinocytes in the epidermis, as its name suggests, but also works on the blood vessels, promoting blood circulation and new blood vessel formation around follicles, which provides nourishment for the growing and differentiating cells [59,68]. In vivo experimental models should be further developed and investigated to fully assess the VEGF-mediated effects of α-phellandrene on hair growth.

## 4. Materials and Methods

### 4.1. Cell Culture

Primary human follicle DPCs (55-year-old, Caucasian, females) isolated from the human dermis of the scalp were purchased from PromoCell (Heidelberg, Germany) and grown in DPC growth medium with supplement mix (PromoCell) and 1% penicillin-streptomycin (10,000 U/mL; Gibco; Grand Island, NE, USA) at 37 °C in a 5% CO_2_ humidified atmosphere. At 70% confluency, the cells were sub-cultured according to the PromoCell subcultivation protocol using the DetachKit (2-[4-(2-hydroxyethyl)piperazin-1-yl]ethanesulfonic acid (HEPES) buffer, trypsin/ethylenediaminetetraacetic acid (EDTA), and trypsin neutralization solution; PromoCell). Passages 3–5 of DPCs were used in this study.

### 4.2. cAMP Measurement

DPCs (20,000 cells/well) were plated in a 24-well plate and incubated with the vehicle (DMSO; Sigma-Aldrich; Seoul, Korea), α-phellandrene (0.1, 1, 5, 10, 50, and 100 μM; Sigma-Aldrich) or forskolin (0.1, 0.5, 1, 5, 10, and 50 μM; Sigma-Aldrich) dose-dependently for 30 min. Subsequently, the cells were washed in ice-cold HEPES buffer and harvested by centrifugation at 1800× *g* at 25 °C for 5 min. After the supernatants were removed, the pellets were extracted with 0.1 M of HCl. Then, the extracts were centrifuged at 15,000× *g* and 25 °C for 3 min, and the supernatants were collected. The amount of cAMP in the supernatants was measured using a direct cAMP enzyme-linked immunosorbent assay (ELISA) kit (Enzo Life Sciences; Farmingdale, NY, USA) according to the manufacturer’s protocol. The cAMP concentration in each sample was normalized to the total protein concentration measured using the Bradford assay (Bio-Rad; Hercules, CA, USA).

### 4.3. WST-1 Assay

DPCs (4000 cells/well) were seeded in a 96-well plate, cultured in medium for 24 h, and treated with vehicle (DMSO) as a control, 6.25, 12.5, 25, 50, and 100 μM of α-phellandrene dose-dependently, or 10 μM of forskolin. After 24, 48, or 72 h of treatment, the cells were incubated with the WST-1 solution (Sigma-Aldrich) diluted 1:10 in culture medium for 3 h. The absorbance was measured at 450 nm against a blank (without cells) using an M200 microplate reader (Tecan; Männedorf, Switzerland). To investigate the mechanism underlying the proliferative effect of α-phellandrene, adenylyl cyclase inhibitor SQ22,536 (50 μM, Sigma-Aldrich), PKA inhibitor H89 (10 µM, Sigma-Aldrich), or CREB inhibitor 666-15 (100 nM; Selleckchem; Houston, TX, USA) was added 1 h before 12.5 μM of α-phellandrene treatment. The proliferation percentage of cells was relatively determined by considering vehicle-only treated cells (control) as 100%.

### 4.4. Lactate Dehydrogenase (LDH) Assay

To assess α-phellandrene-induced cytotoxicity, the release of LDH into the culture supernatant was determined using an EZ-LDH assay kit (DoGenBio; Seoul, Korea) in accordance with the manufacturer’s instructions. Briefly, DPCs (4000 cells/well) were seeded in a 96-well plate, cultured in the medium for 24 h, and treated with various concentrations of α-phellandrene (12.5–1200 μM) for another 72 h. The supernatant medium (10 μL) for each sample was then transferred to a 96-well plate and incubated with the LDH reaction mixture (100 μL) for 1 h at 25 °C. To quantify the LDH concentration in the culture media, the absorbance (450 nm) was measured on an Infinite M200 microplate reader (Tecan).

### 4.5. RNA Isolation, Complementary DNA (cDNA) Synthesis, and Quantitative Polymerase Chain Reaction (qPCR)

DPCs (100,000 cells/well) were plated in a 6-well plate and cultured with vehicle or 12.5 μM of α-phellandrene for 6 h. When required, they were pretreated with SQ22,536 for 1 h before α-phellandrene treatment. The cells were harvested for RNA extraction using TRIzol reagent (Invitrogen; Carlsbad, CA, USA) according to the manufacturer’s protocol. In brief, the samples were treated with chloroform (20% of the final volume, Sigma-Aldrich), vortexed briefly, incubated at 25 °C for 5 min, and centrifuged at 12,000× *g* and 4 °C for 15 min. The aqueous phase was transferred to a new tube and an equal volume of isopropanol (Sigma-Aldrich) was added. The samples were incubated at 4 °C for 10 min and centrifuged at 12,000× *g* and 4 °C for 10 min. After the supernatants were removed, the pellets were washed in 70% ethanol, dried, and resuspended in nuclease-free water. Total RNA concentration was measured using a NanoDrop 2000 (Tecan), and cDNA was synthesized using SuperScript IV reverse transcriptase (Invitrogen) and oligo(dT) primer (Invitrogen) on a GeneMax thermal cycler (BIOER; Hangzhou, China). The cDNA was then used for qPCR with gene-specific primers using SYBR green supermix (Bio-Rad) on a CFX Connect Real-Time PCR Detection System (Bio-Rad). qPCR reactions were run at 57 °C annealing temperature for all primers. Glyceraldehyde 3-phosphate dehydrogenase (GAPDH) was used as the housekeeping gene for normalization. The primer sequences used for qPCR are presented in Supplementary Table S1.

### 4.6. Western Blot

DPCs (100,000 cells/well) were seeded in a 6-well plate and incubated with vehicle or 12.5 M of α-phellandrene in a time-dependent manner (0.5, 1, and 3 h). On the contrary, to investigate the effect of α-phellandrene on the cAMP signaling pathway, DPCs were preincubated with 50 μM of SQ22,536 for 1 h, treated with 12.5 μM of α-phellandrene, and further cultured for an additional 0.5 h. Total protein was extracted from the cells using a protein extraction buffer (iNtRON; Seoul, Korea); then, 15 μg of protein (determined by Bradford assay) was loaded onto separate lanes of 8% sodium dodecyl sulfate-polyacrylamide gel and electrophoresed. The proteins were electrotransferred to a nylon membrane (Amersham, Buckinghamshire, UK), and the membrane was blocked with bovine serum albumin (BSA; MP Biomedicals; Auckland, New Zealand) in Tris-buffered saline/Tween-20 (TBST) for 1 h at 25 °C, and incubated overnight at 4 °C with primary antibodies. The primary antibodies used were anti-PKA Cα (Cell Signaling; Herts, UK; 1:1000), anti-GAPDH (Cell Signaling; 1:1000), anti-phosphorylated CREB (pCREB; Cell Signaling; 1:1000), and anti-CREB (Cell Signaling; 1:1000). After incubation with peroxidase-conjugated secondary antibody (Sigma-Aldrich) for 1 h at 25 °C, the binding signals were visualized using an electrochemiluminescence detection reagent (Bio-Rad) on an EZ-capture (ATTO; Tokyo, Japan) and quantified using the ImageJ program (National Institutes of Health; Bethesda, MD, USA).

### 4.7. Quantification of VEGF Levels

DPCs (100,000 cells/well) were seeded in a 6-well plate and cultured with vehicle or 12.5 μM of α-phellandrene. When necessary, they were pretreated for 1 h with SQ22,536 before α-phellandrene treatment. After 48 h, the cell supernatants were collected, and the content of VEGF in the supernatants was measured using Human VEGF ELISA kit (Biolegend, CA, USA) with an M200 microplate reader (Tecan). The final VEGF levels were normalized to the total cellular protein content using the BCA Protein Assay Kit (Takara, Kyoto, Japan).

### 4.8. Statistical Analysis

Data are presented as mean ± standard error of the mean (SEM). The experimental conditions were compared using the two-tailed unpaired Student’s *t*-test using SPSS Statistics 26 (SPSS; Chicago, IL, USA). *p* < 0.05 was considered statistically significant.

## 5. Conclusions

In summary, we have demonstrated, for the first time, that α-phellandrene-mediated increased cAMP levels can enhance proliferation and elevate VEGF levels in DPCs. These findings not only highlight the essential role of cAMP in proliferation and VEGF regulation in DPCs, but also indicate the great potential of α-phellandrene for further developing hair loss treatments.

## Data Availability

The data that support the findings of this study are available from the corresponding author upon reasonable request.

## Supplementary Materials

The following are available online at https://www.mdpi.com/article/10.3390/ijms23168959/s1.
