# Supplementary material for: Activation of cAMP Signaling in Response to α-Phellandrene Promotes Vascular Endothelial Growth Factor Levels and Proliferation in Human Dermal Papilla Cells
Source: Int J Mol Sci. 2022 Aug 11;23(16):8959. doi: 10.3390/ijms23168959 (PMC9409021; doi:10.3390/ijms23168959)
Supplement: Supplementary file 1 [file ijms-23-08959-s001.zip › ijms-1838752-supplementary.pdf]

**Table S1.** Primer sequences used for quantitative polymerase chain reaction (qPCR).

| Gene name | Primer sequences (5'→ 3') |
|---|---|
| Bone morphogenetic protein 1 (*BMP1*) | F: CAGTTTGACTTCTTTGAGACAGAGGGC<br>R: TGTGAGTCCACTGCGCACCTCCACG |
| Colony Stimulating factor 1(*CSF1*) | F: ATGACAGACAGGTGGAACTGCCAG<br>R: TCACACAACTTCAGTAGGTTCAGG |
| Fibroblast growth factor 1 (*FGF1*) | F: ACCGAGAGGTTCAACCTGCC<br>R: GCCATAGTGAGTCCGAGGACC |
| Fibroblast growth factor 11 (*FGF11*) | F: GGCATCGTCACCAAACTGTT<br>R: GCAGTCCCTCAGCATTCATG |
| Glial cell derived neurotrophic factor (*GDNF*) | F: CCCCAGCCATCCAGTCATTC<br>R: ATCGCACTGCCAAGGTTCTC |
| Glucose-6-phosphate isomerase (*GPI*) | F: AGGCTGCTGCCACATAAGGT<br>R: AGCGTCGTGAGAGGTCACTTG |
| Heparin binding epidermal growth factor-like growth factor (*HBEGF*) | F: ATCGTGGGGCTTCTCATGTTT<br>R: TTAGTCATGCCCAACTTCACTTT |
| Insulin-like growth factor 1 (*IGF1*) | F: ATAGAGCCTGCGCAATGGAATAAAG<br>R: AGAAATCCAGAGAGATGGGAGATG |
| Inhibin subunit beta A (*INHBA*) | F: ACACAACAACTTTTGCTGCC<br>R: TCGTGTCACCACTGTCTTCTC |
| Notch ligand jagged-1 (*JAG1*) | F: TCGGGTCAGTTCGAGTTGGA<br>R: CGTTCACGTTCTGCATGGAC |
| Midkine (*MDK*) | F: CGCGGTCGCCAAAAAGAAAG<br>R: TACTTGCAGTCGGCTCCAAAC |
| Nerve growth factor (*NGF*) | F: ATACAGGCGGAACCACACTC<br>R: TGCTCCTGTGAGTCCTGTTG |
| Neurotrophin 3 (*NTF3*) | F: CAGAACATCACGGCGGAAAC<br>R: ACAGACTCTCACTGTCACATACC |
| Neuregulin 1 (*NRG1*) | F: CCGCAGCATCACTTCACACAAAG<br>R: AAGTGGATGGAACCACAGAAAGG |
| Platelet derived growth factor C (*PDGFC*) | F: CTCCTGGTTAAACGCTGTGG<br>R: TATCCTCCTGTGCTCCCTCT |
| Secreted phosphoprotein 1 (*SPP1*) | F: TTCTGATTGGGACAGCCGTG<br>R: TCTCATCATTGGCTTTCCGCT |
| Transforming growth factor beta 1 (*TGFB1*) | F: AGCGACTCGCCAGAGTGGTTA<br>R: GCAGTGTGTTATCCCTGCTGTCA |
| Vascular endothelial growth factor (*VEGF*) | F: CTTCTGAGTTGCCCAGGAGA<br>R: GGATGGAGGAAGGTCAACCA |
| Glyceraldehyde 3-phosphate dehydrogenase (*GAPDH*) | F: AAATCAAGTGGGGCGATGC<br>R: AGGGGGCAGAGATGATGACC |

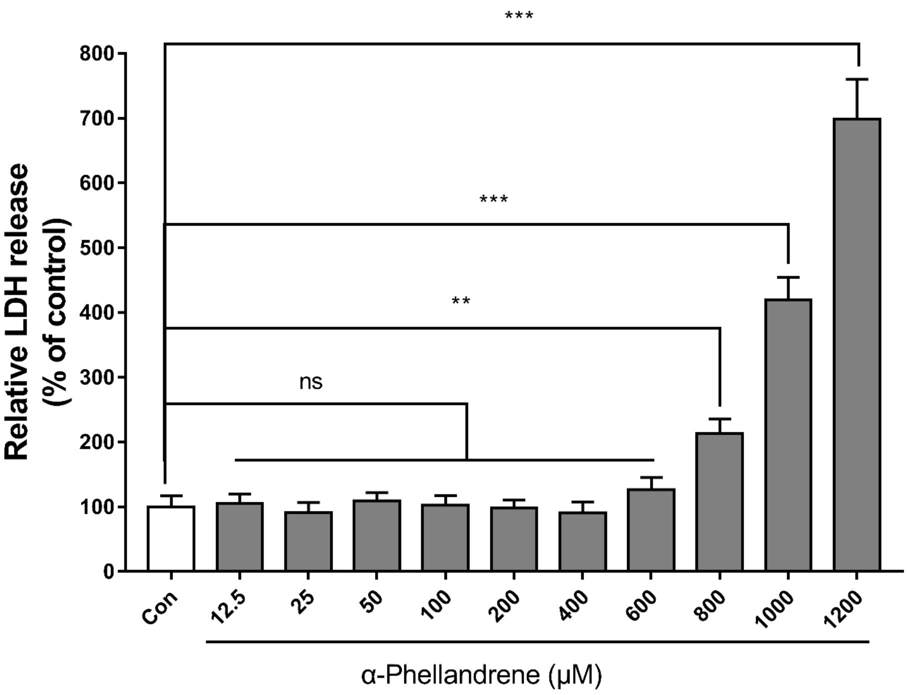


**Figure S1.** $\alpha$-phellandrene up to 600 μM does not induce cytotoxicity in dermal papilla cells (DPCs). DPCs were cultured for 72 h with vehicle (dimethyl sulfoxide; DMSO) as a control (Con), or various concentrations of $\alpha$-phellandrene (12.5-1,200 μM). The cell death in response to $\alpha$-phellandrene treatment was analyzed by lactate dehydrogenase (LDH) assay. Results are shown as mean ± standard error of the mean (SEM) of three independent experiments. Statistically significant differences are marked as ** $p < 0.01$ and *** $p < 0.001$ vs control (Con).
